# Endobronchial High-Dose-Rate Brachytherapy for Posttransplant-Associated Bronchial Stenosis: Extended Follow-up of Pulmonary Function and Subsequent Procedures

**DOI:** 10.1016/j.adro.2026.102159

**Published:** 2026-09-11

**Authors:** Dana A. Schaar, Abigail Yohannes, Abigail Groszkiewicz, Uzoma Iheagwara, Bruce A. Johnson, Matthew Schuchert, Fang Li, Christopher Houser, Elangovan Doraisamy, Hayeon Kim, Steven A. Burton, John A. Vargo

**Affiliations:** a Department of Radiation Oncology, University of Pittsburgh, Pittsburgh, Pennsylvania; b Division of Pulmonology, Department of Medicine, University of Pittsburgh, Pittsburgh, Pennsylvania; c Department of Cardiothoracic Surgery, University of Pittsburgh, Pittsburgh, Pennsylvania

## Abstract

**Purpose:**

Bronchial obstruction is a significant complication after lung transplantation. First-line treatments include a variety of bronchoscopic interventions. As an additional option, there is promise in use of endobronchial brachytherapy for treatment-refractory stenotic areas. We hypothesize that high-dose-rate (HDR) brachytherapy is an effective therapy for treatment-refractory bronchial stenosis and present our institutional data on this approach.

**Methods and Materials:**

This is a retrospective review of double lung transplant patients who underwent endobronchial HDR brachytherapy from February 2005 to July 2025 for symptomatic, treatment-refractory bronchial stenosis. HDR brachytherapy was delivered via flexible bronchoscopy and insertion of a single-lumen brachytherapy catheter. Doses used were 3 or 7 gray (Gy) prescribed to 1 cm depth. Patients were followed with bronchoscopies and pulmonary function testing. Patient, treatment, and outcome data were analyzed descriptively. Percentage predicted forced expiratory volume at 1 second and intervention frequency data were compared with 2-tailed paired *t* tests.

**Results:**

Eight patients were included, comprising 10 brachytherapy treatments, with a median follow-up time of 72.9 months (IQR, 44.8-85.3). Most patients received 1 course of brachytherapy (62.5%), and most treatments were done with 3 Gy in 1 fraction (Fx) (60%). The remainder of patients received 2 brachytherapy treatments (37.5%), and the remainder of treatments were done with 7 Gy in 1 Fx (40%). Median time to next bronchoscopic intervention after brachytherapy was 5.8 months (IQR, 4.3-6.6). After brachytherapy, patients required significantly fewer interventions in the 6 months posttreatment than in the 6 months pretreatment (*P* = .006, median number of interventions 8.5 vs 2.5). There was 1 possible acute toxicity 2 months after treatment and 1 severe late toxicity 22 months after treatment, both in patients who received 2 brachytherapy treatments with higher doses per Fx. There were no second malignancies and no significant change to percentage predicted forced expiratory volume at 1 second posttreatment (*P* = .25).

**Conclusion:**

Low-dose, single-Fx endobronchial HDR brachytherapy is a safe and effective therapy option for treatment-refractory benign endobronchial stenosis after double lung transplant. Treatment with higher doses per Fx and retreatment should be used with caution as they may increase risk of acute and late toxicity.

## Introduction

Bronchial obstruction is a common and clinically significant complication after lung transplantation, with an incidence as high as 21% in the early postoperative period and 70% chronically.^1^ The pathogenesis is multifactorial, believed to start with intraoperative bronchial ischemia that is subsequently compounded by postoperative factors such as infection, immune-mediated injury, and prolonged mechanical ventilation use.^1^, ^2^, ^3^ After the procedure, prevention strategies are introduced early and focus on bronchoscopic surveillance and maintenance immunosuppression.^1^^,^^3^ In the event of early airway complications, interventions such as balloon dilation, stenting, cryotherapy, and ablation are delivered via bronchoscopy,^1^^,^^4^^,^^5^ and for the management of chronic bronchial obstruction, modifications to the immunosuppressive regimen are first trialed, with surgical revision or retransplantation reserved for refractory cases.^2^^,^^3^^,^^6^ For patients who fall between these extremes and enter a cycle of repeated bronchoscopies, there is substantial procedural burden and cumulative risk given their immunosuppression needs; thus, strategies that provide durable control and reduce the requirement for repeat procedures are of particular importance. One such management option is endobronchial high-dose-rate (HDR) brachytherapy.

Interest in the use of radiation for nonmalignant etiologies has been growing. Although radiation’s ablative potential has been leveraged for indications such as ventricular tachycardia,^7^ its anti-inflammatory properties have established its role for indications such as keloids, heterotopic ossification, and osteoarthritis.^8^, ^9^, ^10^ Treatment of refractory endobronchial obstruction with HDR brachytherapy similarly fits in this later, anti-inflammatory space. At low doses, radiation has been shown to offer anti-inflammatory effects thought to be mediated by multiple complex interactions within the immune system, including modulation of inflammatory cytokines and macrophage pathways.^11^^,^^12^

The existing data for HDR brachytherapy in posttransplant airway obstruction shows success, but it remains limited. One of the first studies to report its use specifically in this population demonstrated bronchoscopic luminal improvement as a primary endpoint.^13^ Another study similarly reported benefit using bronchoscopic response and symptom improvement.^14^ The next took a more functional approach, measuring airway luminal diameter and pulmonary function indices, and found reduced reintervention rates compared with bronchoscopic management alone.^15^ Growing evidence has since extended these observations to benign endobronchial granulation tissue obstruction more broadly based on use of bronchoscopic patency and symptom scales.^16^^,^^17^ Taken together, these series share commonalities: encouraging evidence but with small patient numbers, heterogeneous outcome measures, and follow-up rarely exceeding 2 years, leaving the durability and magnitude of benefit an ongoing question.

At our institution, we have treated refractory endobronchial obstruction predominantly with 3 gray (Gy) in 1 fraction (Fx) of HDR brachytherapy, which has a biologically equivalent dose similar to popular regimens for treatment of osteoarthritis^8^ and was selected to generate low-dose anti-inflammatory effects. This is in contrast to much higher doses used either intraluminally or externally for malignant obstruction, in which the goal is to create tumoricidal effects that also carry proinflammatory properties.^12^^,^^18^ Here, we present a review of our institutional experience with treatment of refractory benign endobronchial obstruction after lung transplantation with HDR brachytherapy and aim to contribute long-term data to the current evidence base.

## Methods and Materials

This retrospective study was approved by our institutional review board (IRB approval number: PRO13020306). Data from patients who underwent endobronchial HDR iridium-192 brachytherapy from February 2005 to July 2025 for bronchial stenosis after lung transplantation were retrospectively reviewed. Patients were selected for HDR brachytherapy if they had treatment-refractory, symptomatic stenosis. Patients treated for pathologies other than posttransplant fibrosis were excluded.

On the day of treatment, patients underwent a flexible bronchoscopy and insertion of a single-lumen brachytherapy catheter. Intraoperative catheter placement was confirmed in real time by fluoroscopic imaging; early in the series, postimplant imaging and planning was performed with 2-dimensional x-ray technique, while later in the series, there was a transition to the use of computed tomography simulation. Treatment length was determined clinically and included visible stenosis plus a 1-cm margin on either side. Doses of 3 or 7 Gy in 1 Fx were prescribed to this target length at 1 cm depth from the catheter surface. Treatment planning was performed using Nucletron Oncentra (Elekta). Dose and use of short-interval retreatment varied by treating radiation oncologist, with evolution of practice based on clinical experience. Until 2012, the primary treating physician favored a higher dose per Fx and was more likely to deliver a second treatment, whereas the treating physicians after 2012 favored a lower dose per Fx and were more conservative with retreatment. Patients were followed with bronchoscopies and pulmonary function testing by their transplant pulmonology team.

Descriptive statistics were used for patient demographics, treatment characteristics, and treatment outcomes. Because the skewness coefficient for intervention data and percentage predicted forced expiratory volume at 1 second (FEV1) data suggested high skew, median values were reported for these outcomes. To objectively analyze response to brachytherapy, the number of interventions were recorded for each patient. Interventions were defined as therapeutic bronchoscopic procedures performed for management of recurrent airway stenosis, including balloon dilation, stent placement/revision, steroids, ablation, and any other bronchoscopic treatment documented by the treating pulmonology team. Surveillance bronchoscopies performed without therapeutic intervention were not counted. The number of interventions were recorded for 4 distinct time frames: 12 to 6 months before brachytherapy, 6 to 0 months before brachytherapy, 0 to 6 months after brachytherapy to assess early response, and 6 to 12 months after brachytherapy to assess late response. The number of interventions pre- and postbrachytherapy treatment as well as percentage predicted FEV1 data were then compared with 2-tailed paired *t* test (Microsoft Excel, version 2.104). Because multiple comparisons were made, an adjustment to the significance level was performed with the Bonferroni correction, and the significance threshold was set at *P* < .017 (0.05/3 comparisons). All statistical analyses were performed with patients as the unit. For analyses depending on dates, if patients were treated with >1 Fx, calculations were based on the second treatment date.

## Results

Eight patients comprising 10 brachytherapy courses were included in this study, and median follow-up time was 72.9 months (IQR, 44.8-85.3). Patient characteristics are described in Table 1. All patients (100.0%) underwent a double lung transplant for a variety of diagnoses. The most common treated area of stenosis was the mainstem bronchus (62.5%).

**Table 1:** Patient and treatment characteristics

Patient	Age (y)	Biologic sex	Diagnosis	Surgery	Area of stenosis	Dose (Gy)/Fx
1	66	M	IPF	Double lung	R mainstem bronchus, bronchus intermedius	3/1
2	54	M	IPF	Double lung	R bronchus intermedius	3/1
3	41	F	CF	Double lung	L mainstem bronchus	12/4 3/1
4	57	M	IPF	Double lung	R mainstem bronchus	3/1
5	63	M	COPD	Double lung	R bronchus intermedius	7/1 7/1
6	50	F	Eisenmenger	Double lung	R mainstem bronchus	7/1
7	60	F	Emphysema	Double lung	R bronchus intermedius	3/1
8	62	M	Emphysema	Double lung	R mainstem bronchus, bronchus intermedius	3/1

*Abbreviations:* CF = cystic fibrosis; COPD = chronic obstructive pulmonary disease; F = female; Fx = fraction; Gy = gray; IPF = idiopathic pulmonary fibrosis; M = male.

Treatment characteristics are described in Table 1. The majority of treatments at our institution were with 3 Gy in 1 Fx (60%), and the remainder were with 7 Gy in 1Fx (40%). Most patients underwent 1 treatment of brachytherapy (62.5%), and the remainder underwent 2 treatments (37.5%). One patient who underwent 2 HDR treatments had the first treatment done at an outside University with 12 Gy in 4 Fx and then had a second treatment of 3 Gy in 1 Fx at our institution 11 years later. Two patients had 2 short-interval treatments of 7 Gy in 1 Fx completed at our institution with 2.8 months and 1.4 months separating their courses. All patients who received a single brachytherapy treatment were treated with 3 Gy in 1 Fx. The median length of stenosis treated was 4.25 cm (IQR, 4.0-4.5).

**Figure 1:**
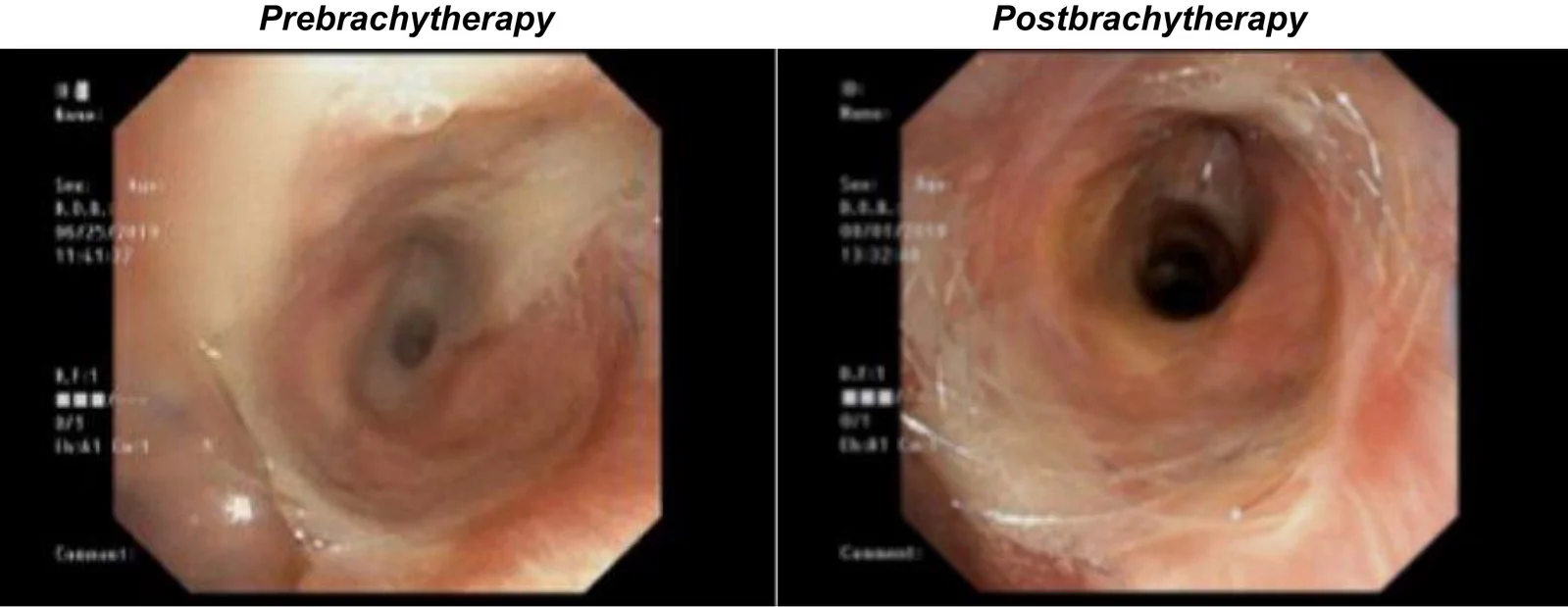
Bronchoscopic images from one patient before and after brachytherapy. Prebrachytherapy image on the left obtained on June 25, 2019, date of brachytherapy was June 28, 2019, and postbrachytherapy image on the right obtained on August 1, 2019.

Median time to intervention after brachytherapy was 5.8 months (IQR, 4.3-6.6). After brachytherapy, patients required significantly fewer interventions in the 6 months after treatment than they did in the 6 months leading up to treatment (*P* = .006), with the median number of interventions per patient in the 6 months before brachytherapy being 8.5 (IQR, 7-9.5) compared with 2.5 (IQR, 0.8-3.5) in the 6 months after brachytherapy treatment. There was also a trend toward fewer interventions in the 6 to 12 months after treatment compared with the 6 to 12 months before treatment (*P* = .08); in the 6 to 12 months before brachytherapy, the median number of interventions per patient was 5.5 (IQR, 2.8-9.3) compared with 0 (IQR, 0-1.8) in the 6 to 12 month period after brachytherapy. Data regarding interventions pre- and postbrachytherapy are detailed in Tables 2 and 3. Additionally, all patients were noted to have bronchoscopic improvement of stenosis after brachytherapy; an example of this is shown in Figure 1.

**Table 2:** Bronchoscopic interventions 0 to 6 months before and after brachytherapy

	0-6 mo pre-HDR						0-6 mo post-HDR						
Patient	Laser	Balloon	Stent	Debride	Steroid	Total	Laser	Balloon	Stent	Debride	Steroid	Total	Difference
1	4	5	2	0	0	11	1	1	0	1	0	3	−8
2	2	2	0	0	0	4	1	1	0	0	0	2	−2
3	2	3	3	1	0	9	0	0	0	0	0	0	−9
4	2	6	0	0	0	8	1	0	0	0	0	1	−7
5	4	12	1	5	0	22	0	5	0	0	0	5	−17
6	4	2	1	0	0	7	2	0	0	0	1	3	−4
7	3	6	0	0	0	9	3	4	1	0	0	8	−1
8	2	3	0	1	1	7	0	0	0	0	0	0	−7

*Abbreviation:* HDR = high-dose-rate.

**Table 3:** Bronchoscopic interventions 6 to 12 months before and after brachytherapy

	6-12 mo pre-HDR						6-12 mo post-HDR						
Patient	Laser	Balloon	Stent	Debride	Steroid	Total	Laser	Balloon	Stent	Debride	Steroid	Total	Difference
1	3	7	1	1	1	13	0	0	0	0	0	0	−13
2	2	2	0	0	0	4	0	0	0	0	0	0	−4
3	1	1	0	0	0	2	0	0	0	0	0	0	−2
4	4	8	1	0	0	13	1	0	0	0	0	1	−12
5	0	0	0	0	0	0	0	0	0	0	0	0	0
6	1	1	1	0	0	3	4	3	0	0	2	9	+6
7	2	4	0	0	1	7	0	0	0	0	0	0	−7
8	3	4	0	0	1	8	1	3	0	0	0	4	−4

*Abbreviation:* HDR = high-dose-rate.

All patients tolerated the brachytherapy procedure well without any resulting intraoperative complications or postprocedural admissions. The patient who was treated with 2 HDR treatments of 12 Gy in 4 Fx followed by 3 Gy in 1 Fx had an admission 2 months after treatment for cough and worsening dyspnea requiring an increase in steroid dose; the etiology of this event was not diagnosed, and therefore it is possible it could have been brachytherapy treatment-related. One patient who was treated with 2 treatments of 7 Gy in 1 Fx had a late toxicity 22.5 months after brachytherapy of hemoptysis requiring embolization with interventional radiology; on follow-up bronchoscopy, an area of oozing telangiectasia was seen in the radiation-treated area that was within the embolization field. Data regarding percentage predicted FEV1 is shown in Table 4. Median percentage predicted FEV1 prior to brachytherapy was 59.5% (IQR, 37.8-80.6), and median percentage predicted FEV1 after brachytherapy was 64% (IQR, 38.8-89.6); there was no decline in percentage predicted FEV1 after treatments (*P* = .25). There were no second malignancies noted throughout the follow-up intervals.

**Table 4:** Mean percent predicted FEV1 values before and after brachytherapy

Patient	FEV1 % predicted pre-HDR	FEV1 % predicted post-HDR
1	65	72
2	33	40
3	27	25
4	54	56
5	122	120
6	39	35
7	89	126
8	78	79

*Abbreviations:* FEV1 = forced expiratory volume at 1 second; HDR = high-dose-rate**.**

Of the 8 patients, 4 (50%) had died at the time of this analysis. Median time to death was 55 months (IQR, 36.7-97.8), and for the 4 patients alive at the time of analysis, median follow-up was 78 months (IQR, 54.1-92.1). One patient died of cardiac arrest secondary to pneumonia from immunosuppression in the setting of her double lung transplant. One patient died of acute hypoxia secondary to severe mainstem bronchus stenosis, severe pneumonia, and pulmonary embolism; notably, the stenosis was in a different area than the one treated with HDR. The other 2 patients died of acute renal failure after the decision to not pursue hemodialysis.

## Discussion

Standard management of posttransplant bronchial stenosis often requires repeated therapeutic bronchoscopic interventions which, while effective, are inconvenient for patients and expose them to procedural risks and potential infectious complications in the setting of chronic immunosuppression. Therefore, identifying interventions capable of providing more durable airway patency is clinically meaningful even if they do not completely eliminate the need for future procedures. HDR brachytherapy offers a minimally invasive alternative that can reduce the frequency of interventions by modulating local tissue proliferation. This study of 8 patients with long-term follow-up adds to the accumulating evidence base that supports low-dose-per-Fx HDR brachytherapy as a safe and effective treatment for refractory benign bronchial stenosis in patients after lung transplant.

Kennedy et al^13^ was the first to demonstrate that bronchoscopic patency could be improved with HDR brachytherapy in posttransplant patients, establishing proof of concept. Halkos et al^14^ and Brenner et al^19^ reinforced this signal using similar bronchoscopic endpoints, then Meyer et al,^15^ Tendulka et al,^20^ and Madu et al^16^ went further, incorporating pulmonary function indices and showing reduced reintervention rates, a shift toward more functional, clinically actionable outcomes. Further details about these series are summarized in Table 5.^4^^,^^13^^,^^14^^,^^16^^,^^19^^,^^20^ What this body of work could not fully answer, given their short follow-up and heterogeneous dosing, was whether the benefit was durable and whether it could be achieved safely with a standardized low-dose approach. The present study, with a median follow-up of 72.9 months from 2 dose schemes, addresses both questions.

**Table 5:** Comparison of present study to published series

Author Year	N	Indication	Dose	Short interval Re-tx	Outcomes	Acute toxicity	Late toxicity	Median follow-up(mo)
Present study 2026	8	Posttransplant bronchial stenosis	3 Gy (n = 6) or 7 Gy (n = 2) to 1 cm depth	Yes; n = 2	All with bronchoscopic improvement; reduction in bronchoscopic interventions; median 5.8 mo to next intervention	Possible respiratory exacerbation (n = 1)	Hemoptysis requiring embolization (n = 1)	73
Meyer et al^15^ 2012	12	Posttransplant symptomatic bronchial stenosis	3 Gy to 5 mm depth	Yes; n = 6	All with clinical and functional improvement; reduction in bronchoscopic interventions; improvement in FEV1	None reported	None reported	34
Tendulkar et al^20^ 2008	8	Posttransplant or poststent recurrent bronchial stenosis	7.1 Gy to 1 cm depth	Yes; n = 5	Reduction in bronchoscopic interventions; improvement in FEV1; 75% with good early response, 12.5% with good late response	None reported	Anastomotic dehiscence (n = 1), fatal hemoptysis (n = 1)	14.6
Madu et al^16^ 2006	5	Posttransplant or postprolonged intubation recurrent stenosis causing functional deficit	5 Gy (n = 1) or 7 Gy (n = 4) to 1 cm depth	Yes; n = 5	Reduction in bronchoscopic interventions; improvement in FEV1	Radiation bronchitis (n = 1)	None reported	12
Brenner et al^19^ 2003	6	Recurrent stenosis (n = 6) or preventative (n = 2) after stenting for trauma induced bronchial stenosis	10 Gy to 1 cm depth	No	83% with complete or near-complete airway restoration	None reported	Tissue necrosis in patient with prior EBRT (n = 1)	15
Halkos et al^14^ 2003	4	Posttransplant recurrent bronchial stenosis	3 Gy to 1 cm depth	Yes; n = 4	Variable from poor, with no improvement and death from progressive airway structure, to excellent, with substantial improvement and no further interventions	None reported	None reported	24
Kennedy et al^13^ 2000	2	Posttransplant bronchial stenosis	3 Gy to 1 cm depth	Yes; n = 1	Both with symptomatic improvement maintained at 6 mo	None reported	None reported	6.5

*Abbreviations:* EBRT = external beam radiation therapy; FEV1 = forced expiratory volume at 1 second; Re-tx = retransplantation.

Before further discussion of outcomes, there are a few aspects of endobronchial HDR treatments worth special note and careful consideration when reviewing these cases and prior series. First is the dose used; although the intent of all treatments was delivery of an anti-inflammatory dose, there is a large difference between 3 Gy per Fx and 7 Gy per Fx that is made even more divergent by the heterogeneity of dose distribution when prescribing to 1 cm depth. Second is the placement of the brachytherapy catheter; if the brachytherapy catheter is in contact with the bronchial wall and appropriate prescription adjustments are not made, there would be resulting very-large-dose hot spots that could increase risk for late toxicity. Catheter placement in the present study was not always retrospectively reviewable due to the 2-dimensional planning used early in the study period; more contemporaneously, there was transition to 3-dimensional computed tomography-based planning to allow for better accounting of any variability in catheter placement. Many of the other series discussed above also used 2-dimensional planning techniques and endorsed the possibility of eccentric catheter placement.^13^^,^^16^^,^^19^^,^^20^

The prior studies of HDR brachytherapy for benign airway obstruction discussed above had variability in dose per Fx and in allowment of retreatment, but most utilized either a high dose for a single treatment only or frequently retreated in all or almost all patients.^13^, ^14^, ^15^, ^16^^,^^20^ In contrast, the present study predominantly used a uniform single-Fx dose of 3 Gy. With this dosing regimen, our cohort demonstrated comparable or improved clinical outcomes compared with the prior series, including universal bronchoscopic improvement after treatment, a median time to next intervention of 5.8 months, and a substantial reduction in the frequency of bronchoscopic interventions compared with the pretreatment period. Specifically, there was a significant reduction of 70% in the mean number of bronchoscopic interventions during the 0 to 6 month period after treatment from 8.5 to 2.5. Considering that patients required a median of 8.5 interventions in the 6 months before brachytherapy, which is >1 per month, this reduction to <1 every 2 months represents a substantial decrease in procedural burden and infection risk. Additionally, there was a trend toward fewer interventions during the 6 to 12 month period after HDR brachytherapy, suggesting potential durability of treatment effect. Together, these findings suggest that a standardized low-dose regimen of 3 Gy may achieve similar therapeutic benefit while potentially reducing the risk of radiation-related toxicity.

Two of the principal concerns with endobronchial HDR for benign bronchial stenosis are the risk of radiation-induced malignancy and potential decrements in already precarious lung function. Therefore, the lack of percentage predicted FEV1 decline and lack of secondary malignancies in this cohort both point toward the overall safety of HDR brachytherapy in this population of patients who are postlung transplant. However, our data also suggests that there may be increased risk with use of 2 treatment courses, especially with higher doses per treatment. Acute toxicities of HDR endobronchial brachytherapy in the postlung transplantation setting are typically transient and mild, most often presenting as cough, mild bronchitis, or short-term worsening of baseline respiratory symptoms, whereas cases of pneumonia and pneumothorax occur less frequently.^17^ In this study, there was 1 patient who had a potential acute toxicity of admission for cough and dyspnea 2 months after a second brachytherapy treatment with 3 Gy in 1 Fx after previous treatment with 12 Gy in 4 Fx. For this population, who are prone to respiratory illnesses and symptoms due to their transplant and immunosuppressed status, it can be hard to delineate what respiratory symptoms in the posttreatment period are secondary to treatment versus the nature of their disease. Therefore, given there was no definitive diagnosis for this patient at discharge, the case was included as a potential toxicity from brachytherapy to err on the conservative side.

For late toxicity after endobronchial brachytherapy, radiation-induced bronchitis, airway necrosis, fistula formation, bronchial stenosis, and rarely, fatal hemoptysis are of concern. The most feared late toxicity, fatal hemoptysis, occurs at a rate of 5% to 10% in malignant cases, although it appears to be substantially less common in benign, posttransplant settings when conservative dosing and careful patient selection are utilized.^17^^,^^21^ Higher cumulative dose, larger irradiated volume, repeat irradiation of the same site, and prior external-beam radiation have been identified as risk factors for late toxicity in the malignant application.^22^ Though fortunately not fatal, one patient had a massive hemoptysis secondary to radiation-induced telangiectasia that could have been fatal without effective intervention of intubation and embolization. The patient who experienced this toxicity was treated with 2 treatments of 7 Gy separated by 2.8 months and had hemoptysis occur almost 2 years after the second treatment. As mentioned above, this patient was treated early in the series when 3-dimensional imaging was not obtained, and therefore it was not possible to check for correct catheter placement in the retrospective review. Certainly, if any deviation of the catheter toward the bronchial surface occurred, this could have potentially contributed to this event and been further exacerbated by prescription of a high dose to depth.

Retreatment and higher dose per Fx in other series were common, and the majority did not report resulting late toxicity; however, the follow-up was short, <2 years in most series.^13^, ^14^, ^15^, ^16^^,^^19^ Given the late toxicity in the present report happened after ∼2 years and would have occurred outside of these follow-up periods, it is possible that prior series would have shown late events with prolonged follow-up. Tendulka et al^20^ utilized both high dose per Fx and frequent retreatment, which did result in 2 late toxicities, one of which was also a hemoptysis event that unfortunately was fatal. The patient that experienced this toxicity was treated with 7 Gy × 2 Fx, and the event occurred 5 months after treatment. Taking these accounts into consideration, together with the present series in which 2 of 3 patients receiving multiple HDR treatments with higher doses per Fx experienced toxicity, suggests that higher dose per Fx and retreatment may carry increased risk and therefore should be used thoughtfully and with caution.

Study limitations include its retrospective design, small sample size, and variability in dose regimens. Additionally, the study included 3 patients who were treated with 2 separate brachytherapy treatments, which were delivered as close as 42 days apart and as far as 11 years apart. This introduces heterogeneity into this small cohort of patients and makes it difficult to draw definitive conclusions about the safety of retreatment. Third, due to electronic health records undergoing a major transformation during the study period, documentation about some details regarding treatment decisions are lacking.

Future research could include exploration of patient-reported outcomes to better define the role of HDR brachytherapy in the patient experience. A randomized trial would also provide further evidence regarding efficacy in comparison to other bronchoscopic interventions.

## Conclusion

Endobronchial HDR brachytherapy delivered using low-dose single-Fx regimens is a safe and effective treatment for refractory bronchial stenosis after double lung transplantation that resulted in significantly fewer bronchoscopic interventions after treatment, demonstrated bronchoscopic improvement of stenosis, and maintained pulmonary function without resulting secondary malignancy. Use of higher dose-per-Fx regimens and retreatment with a second brachytherapy should be used with caution as it may put patients at higher risk for acute and late toxicities.

## Disclosures

None.
